# Targeted health promotion with guided nature walks or group exercise: a controlled trial in primary care

**DOI:** 10.3389/fpubh.2023.1208858

**Published:** 2023-08-24

**Authors:** Annika Kolster, Malin Heikkinen, Adela Pajunen, Anders Mickos, Heini Wennman, Timo Partonen

**Affiliations:** ^1^Department of General Practice and Primary Health Care, University of Helsinki, Helsinki, Finland; ^2^Health Services, Western Uusimaa Wellbeing Services County, Espoo, Finland; ^3^Health and Social Welfare Service, Eastern Uusimaa Wellbeing Services County, Sipoo, Finland; ^4^Luonnontie, Helsinki, Finland; ^5^Primary Health Care Unit, Helsinki University Hospital, Helsinki, Finland; ^6^Department of Public Health and Welfare, Finnish Institute for Health and Welfare, Helsinki, Finland

**Keywords:** green space, mental wellbeing, sleep, primary care, social prescribing, exercise, nature-based interventions, health promotion

## Abstract

**Background:**

Contact with nature promotes wellbeing through diverse pathways, providing a potential way of supporting health especially in primary care, where patients commonly suffer from multimorbidity and poor general health. Social prescribing is a non-pharmaceutical approach for improving health as well as social inclusion. This field study explores and compares the effects of a nature-based and an exercise-based social prescribing scheme on mental wellbeing and sleep, in a primary care population.

**Methods:**

Primary care patients identified to benefit from a general improvement to their health were recruited by nurses, doctors, or social workers to this non-randomized, intention-to-treat, pilot field-study. Participants (*n* = 79) chose between the group interventions, either taking part in guided walks in nature, including immersion in a forest with high biodiversity, or participating in a versatile sports program. Mental wellbeing was assessed with the Warwick-Edinburgh Mental Well-Being Scale (WEMWBS), with additional questions evaluating self-rated health and sleep. Impact on mental wellbeing was explored in relation to perceived health. The amount and quality of sleep was measured with wrist-worn accelerometers. With a focus on everyday life impacts, the assessments took place before and after the 8-week intervention. All participants lived in Sipoo, Finland, an area with abundant accessible green space.

**Results:**

Participants (mean age 57 years, 79% female) rated their general and mental health lower than the general population. Participation in the Nature-group resulted in improved mental wellbeing (change in WEMWBS by 3.15, *p* = 0.008), with a positive change for feeling relaxed, being cheerful, having energy to spare, feeling able to deal well with problems, feeling good about oneself and feeling close to other people. The Sports-group was beneficial for those initially rating their health as good. Sleep duration improved in the Sports-group, while participants in the Nature-group reported better sleep quality. Following the interventions there was improvement in perceived health and ability to function in both groups, while perceived mental health improved only in the Nature-group.

**Conclusion:**

We attest that even in areas surrounded by greenery, active interventions can further improve health in a primary care population, and that nature-based interventions are beneficial for those in poor health.

**Clinical trial registration:**

ClinicalTrials.gov, Identifier NCT05893212.

## 1. Introduction

Managing and increasing accessible green spaces has been recommended as a potential way of improving public health (1, 2), while simultaneously responding to the loss of biotopes and biodiversity in a world that is rapidly becoming more urban (3–5). However, how and when nature could be utilized as a treatment for specific diseases or health issues is unknown (2, 6–8). As the natural environment is a varied concept, the type of biotope and the amount of biodiversity, green or blue space, as assessed with the normalized difference vegetation index (6), as well as the type of activity undertaken, must be considered when examining health outcomes (9). Using nature-based interventions as a part of medical treatment and rehabilitation is an evolving and potential method of improving both individual and public health (8). Nature-based social prescribing, also called green social prescribing, allows health professionals to refer clients or patients with particular needs to facilitated nature-based intervention, often in groups (10, 11).

Studies exploring the effects of nature-based social prescribing are still uncommon (8). Growing evidence supports the use of nature-based interventions in improving mental health in adults based in community, but there is less evidence on improved physical health (8, 12). When prescribing nature-based interventions we need to be explicit about who they benefit, as well as how and when, especially if interventions are funded and provided by national health systems. There is a lack of studies assessing patients with multiple morbidities, i.e., those with two or more chronic diseases or sense impairments (6, 13). Multimorbidity is common in those utilizing primary care, especially among frequent users (14). Multimorbidity is known to reduce quality of life and mental wellbeing (15), it has been recognized that there is a need to develop effective interventions improving outcomes (13). There is an increasing understanding that perceived health and positive mental well-being are independent predictive factor for health outcomes (16, 17).

Healthier sleep is associated with better mental wellbeing (18), making sleep an important factor to consider when studying effects of nature-based interventions on mental health. Poor sleep correlates with chronic pain and prolonged sick leave (19), both of which are common presentations in primary care. Insomnia is a condition that is overlooked but it increases the risk of adverse health outcomes and the use of potentially harmful drugs (20), while difficulty falling asleep is associated with increased all-cause mortality in middle-aged and older populations (21).

Our study aims to:

1. explore and compare the effects of a nature-based social prescribing scheme and an exercise-based social prescribing scheme on mental wellbeing in a primary care population,
2. analyze general and mental health outcomes as well as sleep characteristics in a primary care population participating in social prescribing schemes, and
3. test if the effects on mental wellbeing of two different social prescribing schemes are different based on participants general health or mental health status.

## 2. Methodology and material

### 2.1. Study design

This is a controlled pilot field-study on parallel groups in an intention-to-treat setting. Participants were recruited from the population using the Health and Social Service Centre of Sipoo and all live in the municipality of Sipoo. Although situated only 17 km from the city center of Helsinki, the capital of Finland, Sipoo is a rural area with abundant green and blue spaces. The population density is 65 persons/km^2^ (22). There is an active political will in Sipoo encouraging the use of green spaces for recreation, and the accessible national park of Sipoonkorpi lies in the area. It is likely that participants in the study spend time outdoors and they have easy access to the natural environment. The air quality in the area is generally very good (23). Participants were recruited among primary care patients by nurses or medical doctors, or among social service clients by social workers. Enrolment was not based on a diagnosis, but staff were instructed to identify clients they felt could benefit from a targeted health intervention and to involve in particular those in poor general health. The project was also described in the local media. Adults who were able to slowly walk approximately 2 km could join the study. All the participants were referred to the study by health professionals and were provided information on the groups before choosing between either the Nature-group or the Sports-group. Although the reason to refer was not reported, enrolment in either intervention was a part of real-life treatment.

The intervention program adapted in this study was developed during the Terveysmetsä (transl. Health Forest) project, a national network project established in 2014 (24, 25). Since 2015 two nature guides, who are not medically trained, have organized 8-week rehabilitation programs for patients who frequently visit the health center in close co-operation with a team of doctors and nurses. The positive response gave rise to a need for a more systematic evaluation on the impact of the program, resulting in this study. The living premises were the same for both groups and did not change through the study.

The Nature-group program included learning more about local outdoor areas and nature itself, the biotopes visited were chosen to provide varied nature experiences, including forests, farmland, lakes, and seashore. Accompanied by nature guides the group practiced simple sensory exercises that enhance contact with nature and its microbiome. A more detailed program is attached in Supplementary Data.

The Sports-group participated in an exercise program and met weekly in community sports facilities. Exercise we define as a planned, structured, repetitive, and purposeful form of physical activity that aims to improve or maintain one or more components of physical fitness. The sports program was planned and executed by professional sports instructors in cooperation with health professionals and included both aerobic and anaerobic exercise as well as team sports. The content of the exercise program was planned according to current best practice and considering the participants physical ability. Details of the program are attached in Supplementary Data.

The intensity and duration of the physical activity as well as the social interaction were designed to be as alike as possible in the compared groups. Both groups were offered a meal or snack during or after the session, with the aim of increasing cohesion. The intervention was free of charge, but travel expenses (mostly by car or bus, <10 km) were not covered. With a maximum of 20 participants the groups met 7 times during an 8-week period. Every session took place in a different location. This enabled the participants in the Nature-group to become familiar with the different outdoor areas, and the Sports-group to become familiar with local sports facilities. The planned activity level was modest, equaling approximately 2 km of walking at slow pace. The intervention started in 2018 and was planned to go on until 2020, including 160 participants equally distributed between the groups. The participants were considered to have fulfilled the program if they attended 5 or more sessions. Based on previous studies in similar populations, we emphasized a 25% drop-out. The intervention was completed twice a year (spring and autumn) during the years of 2018 and 2019 which causes some variance as Finland is a northern country with dark, and commonly cold winters. The COVID-19 epidemic in 2020 hindered the group interventions reducing the total number of participants. A Nature-group was organized in autumn 2020, but its data is not included in this study due to the contrasting general circumstances. A separate qualitative follow-up study was conducted in 2022 (26).

It was possible to participate the intervention without being part of the study. Participants in the study signed an informed consent form allowing the use of data and giving permission to recontact. Participants were free to withdraw from the study at any time without giving a reason, and this did not interfere with their care in any way. The study was approved by the coordinating Ethical Committee of Helsinki and Uusimaa Hospital District (HUS/3520/2017) and study permission was granted by the municipality (7.2.2018). All data is anonymized and stored at the Finnish Institute for Health and Welfare (THL).

### 2.2. Methodology

**Figure 1:**
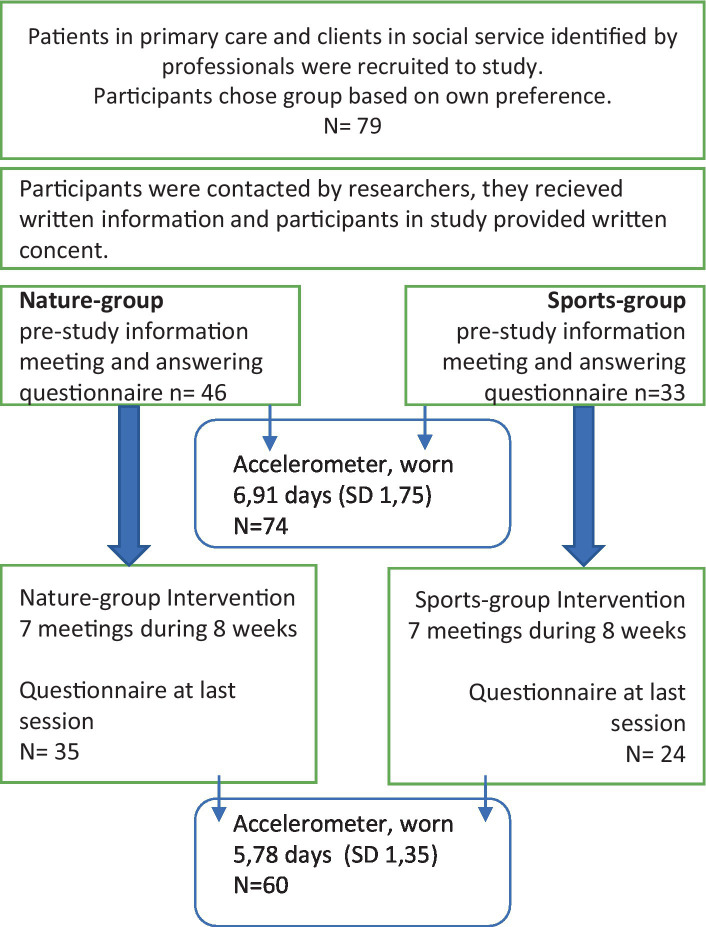
Flowchart of intervention.

Our main outcome is the self-assessed mental wellbeing. Secondarily, we also analyze self-reported and device-based sleep. A flow-chart of the assessments is displayed in Figure 1.

#### 2.2.1. Questionnaire

Self-assessed mental wellbeing was measured with the 14-item Warwick-Edinburgh Mental Well-Being Scale (WEMWBS). The questionnaire also included demographic information (gender, age) and 6 additional questions (Q1–Q6) rated on a Likert-like scale: 1 (little/bad/badly) to 5 (much/good/well). Personal preference was measured in Q1 (*Is nature important to you?*) and Q2 (*Is physical exercise important to you?*). Perceived health and sleep were assessed in questions Q3 (*How is your health at the moment?*), Q4 (*How is your mental health at the moment*?), Q5 (*How is your ability to function at the moment*?), and Q6 (*How do you sleep at the moment*?). Answers were collected before beginning the study, at the fourth meeting, and at end of study. We report the answers from the beginning and the end. Questionnaires were in the native languages Finnish and Swedish. Demographic variables include age and gender. Information on the socioeconomic status, ethnic background, or time of residency in the area were not collected.

The WEMWB scale was developed to assess positive mental wellbeing (27, 28), and although it was not devised to diagnose mental illness, it has shown consistency with scales assessing depression and anxiety disorders (29, 30). The WEMWBS has been validated for use in primary care populations in Nordic countries (31). The WEMWBS measure is responsive to change on both individual and group level (29, 30).

#### 2.2.2. Accelerometer measurements of sleep

Each participant was provided with a wrist worn accelerometer *(ActiGraph GT9X Link, Actigraph LLC, Pensacola, Florida, United States)* to assess their physical activity and sleep before starting the program and after the intervention. Participants were instructed to wear the accelerometer for at least three consecutive days, removing it only if taking a bath or having a sauna. Participants kept a diary of their sleep schedules while wearing the accelerometer. Accelerometer data were extracted and analyzed using Actilife 6 software (*Actigraph LLC, Pensacola, Florida, United States*). For the purposes of this study accelerometer-based sleep periods were analyzed using the Cole-Kripke sleep detection algorithm (32). Analyzed measures included: (1) total sleep time (minutes), (2) time in bed (minutes), (3) sleep efficiency (%), (4) the number of times the participants awoke having already fallen asleep, and (5) length (minutes) of wakefulness after sleep onset averaged over the measurement days.

#### 2.2.3. Statistical analyses

We created groups for self-reported health outcomes, where scores of 1 to 3 were regarded as poor health and scores of 4 and 5 as good health. Data were tested for normality. The differences between the Nature-and Sports-groups as well as between the categorial groups were assessed using Student’s *t*-tests for normally distributed variables and using Pearson’s Chi-squared tests or Mann–Whitney *U*-tests for non-normally distributed variables as appropriate.

The changes in WEMWBS, Q1–Q6 scores and accelerometer data were analyzed using either dependent t-test or Wilcoxon signed-rank test, depending on the distribution of data. Answers of Q1–Q6, gender, age, group, and season were further used as covariates in univariate regression models analyzing factors which may explain the changes in the WEMWBS sleep outcomes. The statistical analyses were performed using IBM SPSS Statistics, version 27, software.

## 3. Results

A total of 79 participants are included in the study, their mean age was 57 years (range 29–81), and the participants were predominantly female (79%). The Nature-group was more popular, with 58% of the participants. At the start of the study, the demographic characteristics, personal preferences for nature and exercise or perceived health did not differ between the groups. The spring and autumn groups are also comparable. Due to the small number of participating men, results are not grouped by gender. Of those starting the program, 72% attended 5 or more sessions and were considered fulfilling the program. Three of those signing up for the intervention did not attend any session and the pre-measurement data on two participants was missing. Included participants attended 5.3 sessions on average (SD 1.6). We tested the likelihood for dropping out of the intervention but found no probability depending on or either the self-rated health, mental health, physical ability, sleep, season, gender, or group.

The accelerometer was worn for 7 days on average (SD 1.76, range 2–9) before the intervention and for 6 days on average (SD 1.38, range 3–8) after the intervention. Descriptive statistics are presented in Table 1.

**Table 1:** Descriptive data at baseline.

		*All*	Naturegroup	Sportsgroup	*p*
Female n (%) Season, attenders in spring		62 (79) 52 (66)	37 (80) 29 (63)	25 (76) 23 (70)	0.618 0.633
	n	M (SD)	M (SD)	M (SD)	
Age in years (Range 29-81) Attendance, n (Range 1-7)	73 75	57 (11) 5,3 (1,6)	58.2 (11) 6 (1,8)	55.6 (11.8) 5 (2)	0.171 0.165
Accelerometer use			n	M (SD)	
Days used before intervention Days used after intervention			74 60	6,91 (1,75) 5,78 (1,35)	

*p* value indicates difference.

### 3.1. General health

At baseline, only 26% of the participants considered themselves in good general health on the 1-to-5 scale (mean 2.97, SD 0.93), 20% felt their physical ability was good (mean 2.77, SD 0.92) and 44% rated their mental health as good (mean 3.31, SD 0.94). Following the intervention, the perceived health improved (mean change 0.4, 95% CI 0.21 to 0.59, *p* < 0.001), the functional ability improved (mean change 0.45, 95% CI 0.28 to 0.62, p < 0.001), and the mental health improved (mean change 0.25, 95% CI 0.07 to 0.43, *p* = 0.008).

The descriptive data and the change in outcomes following the intervention are presented in Table 2. Before starting the study, 88% of all attenders found nature important or very important (mean 4.35, SD 0.7). Even though the perceived importance of nature was high, a further improvement (mean 0.17, 95% CI 0.04 to 0.29, *p* = 0.012) was observed in the Nature-group. Exercise was important to 58% of the participants, and at the end of the study, the importance of exercise had increased (mean change 0.28, 95% CI 0.12 to 0.44, *p* < 0.001).

**Table 2:** Perceived health, mental wellbeing and sleep at baseline for all participants and mean change within groups after intervention Baseline information includes all participants, the groups did not differ at baseline.

Total, baseline			Nature-group				Sports-group			
Perceived health	N	M (SD)	N	Mean Change		*p*	N	Mean Change		*p*
How is your health at the moment? (1–5)	75	2.97 (0.93)	36	0.39 (0.16 to 0.62)		**0.002**	24	0.42 (0.09 to 0.74)		**0.01**
26,7% considered their health to be goodᵝ										
How is your mental health at the moment? (1–5)	75	3.31 (0.94)	36	0.39 (0.16 to 0.62)		**0.002**	24	0.04 (−0.25 to 0.33)		0.77
44% considered their mental health to be good ᵝ										
How is your physical ability at the moment? (1–5)	75	2.77 (0.92)	36	0.47 (0.22 to 0.72)		**<0.001**	24	0.42 (0.2 to 0.63)		**<0.001**
20% considered their physical ability to be good ᵝ										
Total WEMWBS score**	68	48.3 (7.9)	33	3.15 (0.87 to 5.43)		**0.008**	21	0.76 (−1.82 to 3.34)		0.545
Sleep				**M (SD)**	z			**M (SD)**	**z**	
How do you sleep at the moment? (1–5)	75	2.88 (1.14)	36	3.5(1)	z= −3.78	**<0.001** ^†^	24	3.3(1)	z= −1.81	0.07^†^
31% felt they slept well ᵝ				**Mean Change**				**Mean Change**		
Total sleep time (h – min)	74	4.8 h (1.2)	35	−15.4 min (−35.35 to 4.54)		0.13	24	14.15 (−10.65 to 38.94)		0.25
Total time in bed (h – min)	74	5.4 h (1.3)	35	−18.35 min (−40.23 to 3.53)		0.1	24	14.95 (−12.91 to 42.82)		0.28
Sleep efficiency (%)	74	89 (4)	35	0 (−0.98 to 0.99)		0.99	24	0.03 (−1.21 to 1.28)		0.96
Wake after sleep onset (min)	74	34 (13)	35	−2.9 (−6.12 to 0.33)		0.08	24	0.81 (−4.6 to 6.2)		0.76
Number of awakenings after	74	14 (5)	35	−1.4 (−2.78 to −0.01)		**0.05**	24	0.95 (−1.07 to 2.98)		0.34
Average length of awakening (min)	73	2.58 (0.6)	35	0.04 (−0.13 to 0.21)		0.62	24	−0.17 (−0.48 to 0.14)		0.27
Importance of nature and exercise				**M (SD)**	**z**			**M (SD)**	**z**	
Is nature important to you? (1–5)	75	4.36 (0.69)	36	4.5(0.6)	z = −2.12	**0.03** ^†^	21	4.4(0.7)	z= −1.41	0.16^†^
88% considered nature important ᵝ				**Mean Change**				**Mean Change**		
Is exercise important to you? (1–5)	75	3.77	36	0.22 (0.02 to 0.42)		**0.03**	24	0.38 (0.1 to 0.65)		**0.01**
58.7% considered exercise important ᵝ										
Change		**M (SD)**	**M (SD)**				**M (SD)**			
Change in total WEMWBS score**	54	2.2 (6.2)	33	3.2 (6.4)			21	0.8 (5.7)		0.17^‡^

ᵝ (score 4-5) *T-test and ^†^ Wilcoxon (difference within groups) ^‡^ independent T test (difference between groups).

** Warwick-Edinburgh Mental Well-Being. Bold values indicates statistical significance.

### 3.2. Mental wellbeing

The change in mental wellbeing using the WEMWBS score was our primary outcome, we analyzed the impact participation in the interventions had on the whole group, as well as considering the Nature-group and Sports-group separately. The compared groups did not differ at baseline, both groups were normally distributed although at endpoint the range was bigger in the Nature-group (Table 2). The participants fulfilling the interventions (*n* = 54) showed a significant improvement in the total WEMWBS score with a mean change of 2.2 points (*p* = 0.01). However, the change observed in the whole group is mainly due to the good effect of the Nature-group (*n* = 33), where a mean change of 3.5 points (*p* = 0.008) was observed, compared to a mean change of 0.4 point (*p* = 0.75) in the Sports-group (*n* = 21). For the Nature-group, participation in the intervention improved: the feeling of being relaxed, the feeling of having energy to spare, feeling of dealing well with problems, feeling good about oneself, feeling of being close to other people, and feeling of being cheerful. The WEMWBS results are displayed in Table 3. Neither age, gender nor season influenced the change in mental wellbeing. In the univariate models, perceived general health (*p* = 0.005), physical ability (*p* = 0.006) and mental health (*p* = 0.012) all statistically predicted WEMWBS change, whereas self-rated sleep did not.

**Table 3:** Change in positive mental wellbeing.

		All participants *n* = 59–60			Nature-group *N* = 34–35		Sports-group *N* = 23–24	
			Mean change (95% CI)		Mean change (95% CI)		Mean change (95% CI)	
Question score 1–5		Mean (SD)		*p*		*p*		*p*
1	I’ve been feeling optimistic about the future	3.5 (0.8)	0.14 (−0.05–0.33)	0.159	0.23 (−0.02–0.48)	0.073	0 (−0.31–0.31)	1
2	I’ve been feeling useful	3.5 (0.8)	0.07 (−0.13–0.26)	0.497	0.08 (−0.16–0.33)	0.499	0.04 (−0.3–0.38)	0.802
3	I’ve been feeling relaxed	3.1 (0.8)	**0.27 (0.06–0.49)**	**0.015**	**0.31 (0.02–0.6)**	**0.039**	0.22 (−0.13–0.56)	0.203
4	I’ve been feeling interested in other people	3.7 (0.8)	0.13 (−0.07–0.34)	0.197	0.14 (−0.08–0.36)	0.201	0.13 (−0.29–0.54)	0.543
5	I’ve had energy to spare	3 (0.8)	**0.35 (0.13–0.57)**	**0.002**	**0.42 (0.11–0.72)**	**0.009**	0.25 (−0.06–0.56)	0.11
6	I’ve been dealing with problems well	3.2 (0.8)	0.18 (−0.04–0.41)	0.109	**0.31 (0.02–0.6)**	**0.039**	0 (−0.37–0.37)	1
7	I’ve been thinking clearly	3.6 (0.7)	−0.07 (−0.27–0.13)	0.497	0.06 (−0.18–0.29)	0.624	−0.25 (−0.61–0.11)	0.162
8	I’ve been feeling good about myself	3.2 (0.7)	0.14 (−0.05–0.33)	0.159	**0.31 (0.07–0.55)**	**0.014**	−0.13 (−0.43–0.17)	0.377
9	I’ve been feeling close to other people	3.4 (1)	0.3 (0.05–0.55)	0.019	**0.39 (0.08–0.69)**	**0.014**	0.17 (−0.28–0.61)	0.445
10	I’ve been feeling confident	3.4 (0.9)	0.1 (−0.12–0.32)	0.359	0.26 (−0.02–0.54)	0.071	−0.13 (−0.48–0.23)	0.479
11	I’ve been able to make up my own mind about things	4 (0.8)	0.02 (−0.14–0.17)	0.829	0.03 (−0.19–0.25)	0.8	0 (−0.23–0.23)	1
12	I’ve been feeling loved	3.6 (1)	0.07 (−0.12–0.26)	0.484	0.19 (−0.06–0.45)	0.128	−0.13 (−0.43–0.17)	0.377
13	I’ve been interested in new things	3.8 (0.8)	0.17 (−0.02–0.36)	0.077	0.22 (−0.05–0.49)	0.103	0.09 (−0.17–0.34)	0.492
14	I’ve been feeling cheerful	3.6 (0.8)	0.19 (−0.03–0.4)	0.086	**0.39 (0.08–0.69)**	**0.014**	−0.13 (−0.37–0.11)	0.266
	Total WEMWBS score	48.3 (7.9)	**2.22 (0.53–3.92)**	**0.011**	**3.15 (0.87–5.43)**	**0.008**	0.76 (−1.82–3.34)	0.545

Measured on the Warwick-Edinburgh Mental Well-Being Scale (WEMWBS). Bold values indicates statistical significance.

### 3.3. Differences in mental wellbeing by general and mental health status

**Figure 2:**
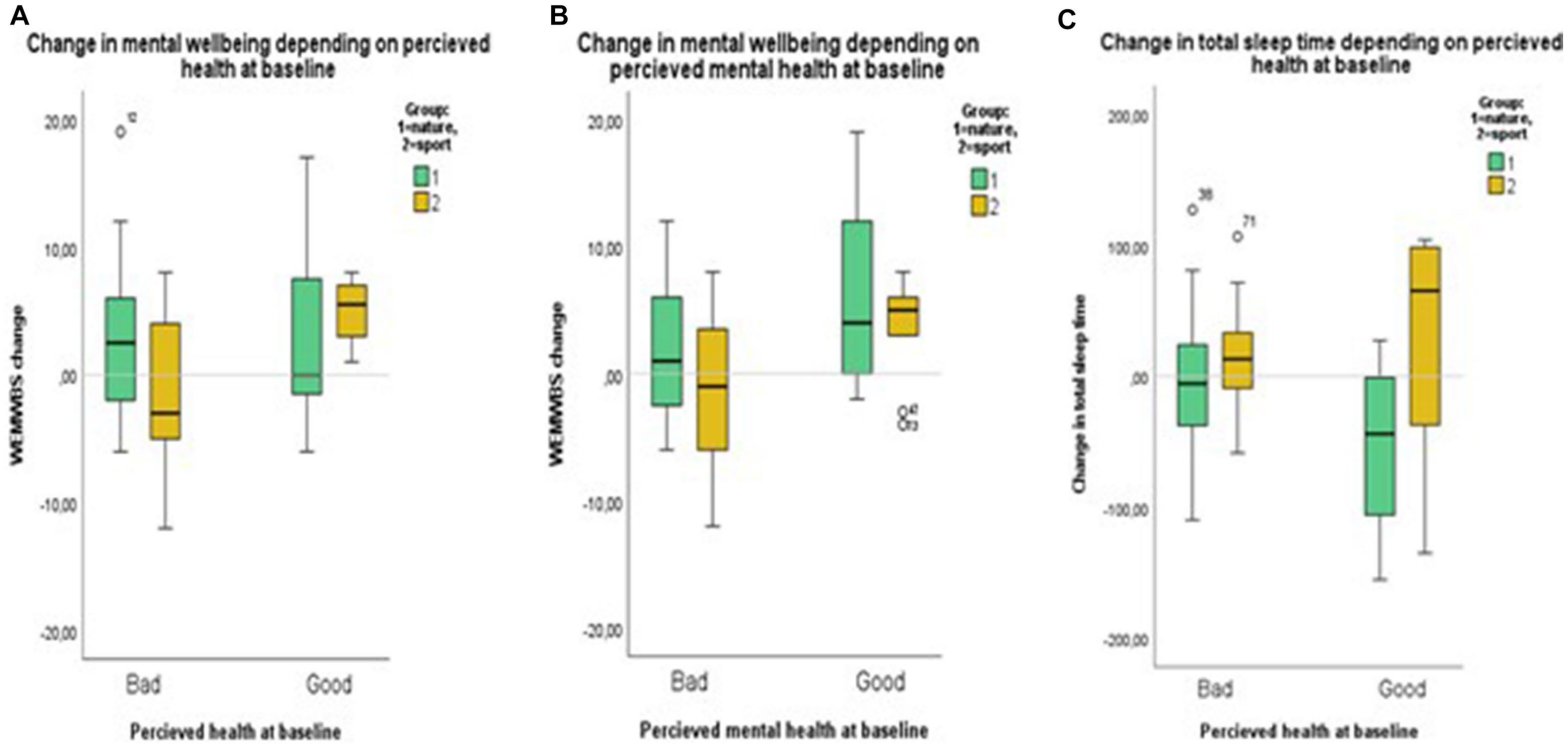
Illustrates change in WEMWBS score depending on **(A)** self-rated health, and **(B)** mental health, as well as **(C)** change in sleep time depending on self-rated health at baseline.

An important finding is that the participants with poor perceived health had less improvement, or even a reduction, in mental wellbeing compared to those considering their health good. Results are presented in Figure 2. In the Nature-group, participants in poor perceived health (*n* = 26) showed an improvement in the WEMWBS scores, the mean change was 3.12 (95% CI 0.77 to 5.46). If health was rated good (*n* = 7), the mean change was 3.29 (95% CI −4.98 to 11.55), the difference depending on the self-rated health status is non-significant (*p* = 0.95). However, in the Sports-group, those in poor health (*n* = 15) showed reduced mental wellbeing, their mean WEMWBS change was-0.93 (95% CI −4.10 to 2.24), whereas those in good health (*n* = 6) had the best response to the intervention, their mean change being 5 (95% CI 2.26 to 7.74), the difference depending on the self-rated health status is significant (*p* = 0.004).

The pattern is similar regarding perceived mental health, and here, there was a difference depending on perceived health in both groups. In the Nature-group, those in poor mental health (*n* = 20) had a mean change of 1.15 (95% CI −1.23 to 3.53) in their WEMWBS scores, but those initially rating their mental health as good (*n* = 13) improved more (mean 6.23, 95% CI 1.87 to 10.6, *p* = 0.039). In the Sports-group, we observed a reduction in WEMWBS scores for participants initially rating their mental health as low (*n* = 12). Their mean change was-1.17 (95% CI −1.99 to 2.65), and if initial self-rated mental health was good (*n* = 9), the WEMWBS scores improved (mean 3.33, 95% CI 0.09 to 6.57, *p* = 0.071).

### 3.4. Sleep

Sleep quality and sleep duration was generally low in this population, only 31% felt they slept well. Mean time in bed assessed by accelerometers was only 5.4 h (SD 1.3) and mean sleep time 4.8 h (SD 1.2). 81% slept less than 6 h per night, usually considered as an insufficient amount of sleep. Accelerometer-based number of awakenings (NOFA) after sleep onset was 14 on average (SD 5) and mean time wake after sleep onset (WASO) was 34 min (SD 13). Sleep efficiency (SE) was 89% on average.

Following the intervention, 61% (22 out of 36) of the participants in the Nature-group experienced a positive change in perceived sleep (z = −3.78, *p* < 0.001). In the Sports-group, 33% (8 out of 24) reported improved sleep quality (z = −1.81, *p* = 0.07). This is contradictory to the observed accelerometer measures, where total sleep time reduced (−15 min) in the Nature-group but increased (+14 min) in the Sports-group. The difference between the groups was near a statistical significance (*p* = 0.06; see Table 2). In the Nature-group, change in total sleep time depended on perceived health. The mean change in sleep time was −48 min (SD 64 min) if health was good (*n* = 9), but-4 min (SD 52 min) if health was rated poor at baseline (*n* = 26). Independent *t*-test indicated a difference between the groups (*p* = 0.047). This pattern was not seen in the Sports-group (Figure 2). In the Nature-group, NOFA decreased by 1.4 on average (95% CI 2.8 to zero, *p* = 0.05) and WASO decreased with 2.9 min on average (−6.1 to 0.3 min, *p* = 0.08), whereas no change occurred in the Sports-group. Sleep efficiency did not change in either group.

## 4. Discussion

First, we address the third aim of this study, i.e., investigating the impacts of health status on the effects of the social prescribing interventions. Next, we discuss about implementation of the interventions, and finally, we discuss on the strengths and limitations of this study.

As intended, participants in the study rated their health lower than the general population. Only 27% regarded their health as good or very good, with the corresponding figures being 62% for men and 63% for women responding to the Finnish population survey FinHealth 2017 (33). Although diagnoses are not reported, we know that multimorbidity is established in the sample. Poor self-rated health is associated with frequent attendance in primary care (34). Health professionals seemed to have a good ability to select those patients suitable for social prescribing programs.

### 4.1. Mental wellbeing

The WEMWBS average of 48 is inferior to that of the general population. In the FinHealth 2017 survey, the average WEMWBS score in southern Finland was 52.8 (95% CI 52.3 to 53.3) (35). Although WEMWBS is not a diagnostic tool, persons with a total score of ≤40 ought to be considered to have a high risk of major depression, while scores between 41 and 45 indicate a high risk of psychological distress and increased risk of depression (17, 28). Several of the participants in this study might have suffered from common mental health disorders, but we lack information on diagnosis or treatment. Most participants in poor health were able to complete the program.

Improved mental health is associated with reduced mortality and mental illness (16), better physical health, social functioning, as well as academic achievement (17, 36). Using the 7-item SWEMWBS scale and applying the English population norms for it, a change of 1 to 3 points may be taken to denote a change that is statistically meaningful (29, 30), therefore the improvement of 3.5 points in the Nature-group observed in our study may be considered as clinically significant. It is of note, however, that we used the 14-item WEMWBS. In the Nature-group, positive changes were observed for the score of feeling able to deal well with problems, feeling good about oneself, and the feeling of being cheerful. This suggests an increase in mental resilience as an effect of nature-based social prescribing and is a significant finding. In the Sports-group, the improvement in mental wellbeing perceived by participants who rated their health as good is also evident, while the slight reduction observed when health was poor is rather not a change. A more pronounced gain in wellbeing for those with better self-rated mental health is common in health interventions. The WEMWBS improvement observed in the Nature-group when perceived health or mental health was poor supports the yet tentative evidence that nature-based social prescribing schemes could be preferrable particularly for persons suffering from mental health disorders (12).

Social interaction and reduced loneliness are potential mediators for well-being (7, 37). Loneliness, a state associated with several adverse health outcomes, is common in all age groups (38, 39). In the Nature-group there was an increase in feeling close to other people. Nature has had a positive effect in empowering groups in previous studies (7, 8). However, some qualitative studies have indicated that people suffering from common mental disorder might react negatively to social pressure, even though the likelihood of visiting green space increased, if experiencing social pressure, the intrinsic motivation and visit happiness might decrease (40). Understanding the underlying cause for individual variance is of importance in future research, perceived mental health might be diminished due to several reasons, i.e., mental disorder or loneliness. In our study, social interaction was planned to be comparable in the groups and both programs aimed to improve social cohesion, thereby equalizing the effect of reduction in loneliness. However, the quality of the social interactions may have differed between the groups, and our results suggest that nature surroundings have played a part in increasing social cohesion and decreasing the experience of loneliness as found in earlier research (41).

### 4.2. Sleep

Poor sleep surfaced as a clear finding, 81% slept less than 6 h per night and 70% of the participants also rated their sleep quality as poor. In the FinHealth 2017 study, the average self-reported sleep time was 7.4 h for adults aged over 30 years, with only 14% of the women and 16% of the men sleeping less than 6 h per night. Following the intervention, total sleep time improved in the Sports-group but decreased in the Nature-group. This change was close to significant and opposite to our expectation. Diminished sleep time was observed only in the participants with good self-rated health. Despite this accelerometer-based outcome, perceived sleep improved significantly in the Nature-group, with reduced time awake after sleep onset and reduced number of awakenings being likely explanations for the positive experience.

Population studies have indicated that surrounding greenery has a positive impact on sleep duration (42), but only a few intervention studies have addressed the issue, with weak study designs (43). Our findings underline the importance of assessing sleep as a part of general wellbeing, and they also show the complexity of insomnia. Time asleep does not always correlate with perceptions of sleep, and stress reduction can partly explain the positive outcomes encountered. Previous studies suggest that residential greenness improves sleep by reducing stress caused by air and noise pollution (42).

### 4.3. Nature connectedness as a potential pathway to positive mental wellbeing

Even though most of the study participants rated nature as very important at baseline, taking part in the Nature-group further strengthened this feeling. Experiencing interconnection with nature increases the motivation to protect and defend it, and the improved connection with nature also increases happiness and wellbeing (44), while reducing anxiety (45). Anxiety and symptoms of depression were not assessed in our study, but previous research indicates nature-based interventions can reduce these symptoms in populations with or without pre-existing mental health problems (12), a strengthened connection with nature is a potential explanation. The Nature-group intervention included activities aiming for increased nature connectedness, as previous research emphasizes the quality of contact with nature rather than mere time outdoors (46).

Contact with nature appears to be important to human health, since living close to green space has an inverse association with all-cause mortality, especially mortality due to cardiovascular disease (2, 4, 47). In addition, contact with nature is known to diversify human microbiome on the skin and in the gut, triggering a healthy immune response (48, 49). The pathways through which exposure to nature influences health are many and interlinked. A widely adopted explanative framework summarizes these pathways by dividing them into those that (a) reduce harmful exposure (air pollution and noise), (b) enhance healthy behavior, and (c) activate human restorative capacities (50). It is likely that these pathways play different roles at different stages through life. Outcomes are also dependent on the way in which we interact in and with our natural surroundings (3).

The restorative capacity of nature is commonly explained by (a) the stress recovery theory, which underlines that contact with nature promotes a positively toned mental state and activates the parasympathetic nervous system (51), or/and (b) the attention restoration theory, emphasizing that nature can restore the ability to direct attention (52). Stress recovery is not only a feeling, but exposure to nature has also repeatedly been demonstrated to reduce physiological stress responses (51, 53). Both healthy and vulnerable populations have shown improved cognitive function during and after outdoor interventions (7, 53, 54). Over time, lack of restoration can lead to mental and physical illness (55).

High perceived stress increases reliance on primary care service (56). Unspecific symptoms secondary to stress, such as anxiety, insomnia or physical symptoms are common reasons to make contact with primary care services (57). In our study, the score for feeling relaxed improved among all participants and significantly in the Nature-group.

There is a knowledge gap regarding what role contact with nature has on an individual level, as personal preferences and cultural values influence how we experience nature and how willing we are to interconnect with it (3). The personal psychological connection with nature is referred to as nature connectedness. Health outcomes vary depending on how we feel in the natural environment (58) and, interestingly, perceived biodiversity can have stronger correlations with well-being than actual biodiversity (48). In national surveys, Finns repeatedly report that they appreciate nature and greenness, and although 72% of Finns live in urban areas, the average distance to a forest is only 700 m (59). Public access rights in Finland allow people to move, pick berries and mushrooms and spend time on public as well as private land, as long as one keeps out of cultivated farmland and private gardens. People living in Finland usually have high nature connectedness and enjoy outdoor activities (59).

### 4.4. Can nature-based social prescribing reduce inequality?

In a global context, Finns are healthy (60). The life expectancy in Finland is among the highest in the world, and Finland has ranked first in global life satisfaction reports, i.e., World Happiness Reports (61). However, health inequity is a big concern, as it is recognized that those who are wealthier are healthier, more physically active and have better access to care than those with lower socioeconomic status (60), even though the Finnish health services are publicly funded and affordable. One concern is that people with risk factors such as sedentary living, obesity, loneliness, or mental disorders do not actively seek help, and are therefore not included in preventive programs. The Finnish Institute for Health and Welfare has estimated that reducing inequalities could reduce direct health-based costs by 1.5 to 2.0 billion euros, where indirect costs due to reduced ability to work are not included in this estimate (62, 63). Gender difference in life expectancy as well as socioeconomic inequality is a well-documented challenge to be solved (62, 64), as women are generally more interested in health-related information and eager to participate in interventions (64). This study was not an exception, the shortage of male participants was a disappointment, we had emphasized that the Nature-group would appeal to men. Hopefully, this will change as we gain more evidence on the effects of nature-based interventions. As a follow-up to the current study, participants of the Nature-group 2018–2020 were contacted during 2022, and 23 of them took part in qualitative interviews reported by Heikkinen (26). Several participants mentioned that they would not have taken part in the intervention had they not been referred by health professionals, and initially thought it was an odd suggestion. Afterwards they found it as a novel and positive part of public health services. The support from the peers and the leaders was also considered very important. Participants also felt they had either established or regained contact with the natural environment and brought up the observation that they had forgotten how meaningful nature is as a source of wellbeing. These themes are in line with theoretical guidance conceptualizing how to best implement nature-based interventions (8).

Primary care patients seek help for various symptoms that may or may not be caused by a disease. General practitioners (GPs) are at the frontline in diagnosing medical conditions, but also familiar with the role that social and psychological factors play in wellbeing. One example is a recent Swedish cross-sectional study of a middle-aged general population concluding that angina pectoris symptoms, irrespective of degree of coronary atherosclerosis, are highly associated with stress and depressive symptoms, among other sociodemographic and psychological factors (65). Approaches for a more holistic health care system are needed. Social prescribing—also called community referral—is an emerging method that enables GPs, nurses, and other healthcare professionals to refer patients in need of support to a range of local, non-clinical services (10, 11). Nature-based social prescribing is a potential way of reducing pressure on health and social service, and although the cost-benefits of social prescribing are yet to be determined, cost savings have been reported when the target group has been frequent attenders (66). Understanding the needs and wishes of those participating in health interventions is crucial in improving motivation and succeeding in behavioral change. In this study, participants in the Nature-group showed improvement in feeling good about oneself, having energy to spare and dealing with problems well, all these qualities being important in order to succeed in change and when coping with chronic disease. Non-pharmaceutical health promotion is an important part of general practice, it is a potential way to prevent illness at an early stage, therefore it is an effective and cost-effective treatment. Although preventative programs are widely used in Finland, they are commonly targeting patients with a specific diagnosis. The concept of social prescribing aiming for a broad improvement in general health and positive mental wellbeing is new in Finland (67).

### 4.5. Strengths and limitations

This is a non-randomized pilot study in a real life, primary care setting and should be interpretated as such. Some aspects can be regarded both as strengths and limitations. First, inclusion was not based on a specific disease or diagnosis, but a common need for general health improvement. This strategic choice is based on the knowledge that primary care patients seek help for various health problems and symptoms, and frequent attenders commonly suffer from symptoms that might not be due to a specific diagnosis (15, 57). Participants referred by social workers may or may not suffer from medical conditions. By choosing an approach aiming to improve health without addressing a medical problem, we lost an opportunity to examine how nature can be used as a treatment for a specific disease. Studies which use diagnoses as outcomes require large population-based samples and long follow-up periods (2) that is beyond the scope of this study. Poor self-rated health is a risk factor for long-term frequent use of primary care (15), but it is unknown to what extent improved perceived health influences the use of health services.

Second, some methodological limitations need to be noted. All participants live in an area with abundant green and blue spaces, therefore, it is likely that the participants in the Sports-group also spent active time outdoors. Sleep was assessed by wrist-worn accelerometers that are considered acceptable for use in population studies or community-based interventions, and they compare rather well with polysomnography, which is considered the gold standard measure of sleep (68). However, there are still weaknesses with accelerometers. They have a low specificity for detecting when one is truly awake (69). The detection of sleep and wake is based on an algorithm which may falsely identify times such as non-wear or motionless periods as sleep, lowering the specificity. Also, it should be noted that if the participants slept during the day, also these periods were included in the averaged sleep data. However, the collected accelerometer data is in line with the self-reported sleep quality measures. To keep the questionnaire simple and to ensure that the participants completed the questionnaire, we preferred single-item questions to multi-item scales for the assessment of nature connectedness and sleep. As 78% of the participants were female, conclusions on the influence of gender cannot be drawn from the study. No adverse events or accidents were reported in either group, but we did not have a structured protocol for collecting information about adverse effects.

Third, the follow-up time is short. The social restrictions following the outbreak of the COVID-19 pandemic ended this study early, therefore, we were able to recruit approximately half of the planned number of participants. A six-month follow-up questionnaire was initially planned, but as the COVID-19 pandemic occupied professionals, it simply was not possible to fulfill this part of the study. Also, we were not able to analyze how participation in the intervention affected the use of health care services. Research in primary care faces pragmatic challenges and needs stronger structure in Finland. Digital tools could facilitate communication between participants and instructors as well as providing a platform for follow-up when developing social prescribing programs. However, consideration of the target groups’ ability to use technical devices is important. The national Sustainable Growth Program in Finland is part of the NextGenerationEU program, aiming to support growth that is ecologically, socially and economically sustainable (70). The Finnish Institute for Health and Welfare is developing models for future healthcare in which social prescribing is included (71). National coordinating is necessary especially in the assessment of the effects of interventions, while field studies like ours provide influential information on the feasibility of the interventions.

Fourth, this was not a randomized controlled trial. The concept of Health Forest started out as a trial, and the study protocol evolved from these experiences. As the project had gained public interest before the study, positive perception may impact the results in favor of the Nature-group, also among the referring health professionals. Even so, the activities for the Sports-group were planned carefully to represent the current best practice. Primary care clients committed to both social prescribing schemes, and no adverse effects nor adverse events occurred. We found no difference in season, and this being found the Nature-groups can be organized all year round also in Nordic countries. Based on the encouraging results we hope to see more preventive projects utilizing nature and research addressing how nature can be used as a treatment. In future research, the use of cluster randomization is a way to provide a more robust study design. In bigger studies, inclusion of health data such as diagnosis and medication could help us understand more about the conditions to which nature-based social prescribing is best of help, and by including information health metrics we might also learn whether nature-based interventions can reduce demand on health and social service.

The programs used in this study can be adapted to different target groups and locations, and we consider the practical approach as the biggest strength of this field-study.

## 5. Conclusion

Our results support the increasing understanding that nature-based interventions have a positive effect on mental wellbeing in primary care patients. In green surroundings, prescribed nature-based interventions or group exercise can improve perceived health and ability to function. Improved mental health and positive mental wellbeing was observed only in the Nature-group. Based on the observed differences in improved mental wellbeing depending on perceived health, we would recommend either sports or nature-activity for those initially feeling healthy, and nature-based intervention especially for those rating their health as poor.

## Data availability statement

The datasets presented in this article are not readily available because according to the research permission all data is anonymised and stored at the Finnish Institute for Health and Welfare. Data can be shared with a specific request from the institute, but not be shared by the researchers. Requests to access the datasets should be directed to research professor TP, timo.partonen@thl.fi.

## Ethics statement

The studies involving human participants were reviewed and approved by Ethical Committee of Helsinki and Uusimaa Hospital District (HUS/3520/2017) and study permission was granted by the municipality of Sipoo (7.2.2018). The patients/participants provided their written informed consent to participate in this study. This study was registered as a clinical trial (ClinicalTrials.gov, Identifier NCT05893212).

## Author contributions

TP, MH, AP, and AM: conceptualization, methodology, and planning. AK, TP, AP, and HW: literature review. HW: preparation of accelerometer data. AK and TP: statistical analysis. AK: writing—original draft preparation. AK, TP, MH, and AP: writing—review and editing. TP: supervision and owner of data. All authors contributed to the article and approved the submitted version.

## Funding

This research was funded by the municipality of Sipoo and the Finnish Institute for Health and Welfare (THL) as well as the Child and Nature Foundation. AK has received research grants from HUS-erva, Perkléns stiftelse, and Finska Läkarsällskapet. Open access funded by Helsinki University Library.

## Conflict of interest

AP was an entrepreneur at Luonnontie, a company developing Health Forest models.

The remaining authors declare that the research was conducted in the absence of any commercial or financial relationships that could be construed as a potential conflict of interest.

## Publisher’s note

All claims expressed in this article are solely those of the authors and do not necessarily represent those of their affiliated organizations, or those of the publisher, the editors and the reviewers. Any product that may be evaluated in this article, or claim that may be made by its manufacturer, is not guaranteed or endorsed by the publisher.

## References

[1] Barboza EP Cirach M Khomenko S Iungman T Mueller N Barrera-Gómez J. Green space and mortality in European cities: a health impact assessment study. Lancet Planet Health. (2021) 5:e718–30. doi: 10.1016/S2542-5196(21)00229-1, PMID: 34627476

[2] Rojas-Rueda D Nieuwenhuijsen MJ Gascon M Perez-Leon D Mudu P. Green spaces and mortality: a systematic review and meta-analysis of cohort studies. Lancet Planet Health. (2019) 3:e469–77. doi: 10.1016/S2542-5196(19)30215-3, PMID: 31777338 PMC6873641

[3] Hartig T Mitchell R de Vries S Frumkin H. Nature and health. Annu Rev Public Health. (2014) 35:207–28. doi: 10.1146/annurev-publhealth-032013-182443, PMID: 24387090

[4] Yuan Y Huang F Lin F Zhu P Zhu P. Green space exposure on mortality and cardiovascular outcomes in older adults: a systematic review and meta-analysis of observational studies. Aging Clin Exp Res. (2021) 33:1783–97. doi: 10.1007/s40520-020-01710-0, PMID: 32951189

[5] World Urbanization Prospects. Available at: https://www.un.org/development/desa/en/news/population/2018-revision-of-world-urbanization-prospects.html: United Nations; (2018).

[6] Geneshka M Coventry P Cruz J Gilbody S. Relationship between green and blue spaces with mental and physical health: a systematic review of longitudinal observational studies. Int J Environ Res Public Health. (2021) 18:9010. doi: 10.3390/ijerph18179010, PMID: 34501598 PMC8431638

[7] Leavell MA Leiferman JA Gascon M Braddick F Gonzalez JC Litt JS. Nature-based social prescribing in urban settings to improve social connectedness and mental well-being: a review. Curr Environ Health Rep. (2019) 6:297–308. doi: 10.1007/s40572-019-00251-7, PMID: 31713144

[8] Masterton W Carver H Parkes T Park K. Greenspace interventions for mental health in clinical and non-clinical populations: what works, for whom, and in what circumstances? Health Place. (2020) 64:102338. doi: 10.1016/j.healthplace.2020.102338, PMID: 32838901

[9] Kim E Park S Kim S Choi Y Cho J Cho SI. Can different Forest structures Lead to different levels of therapeutic effects? A Systematic Review and Meta-Analysis. Healthcare. (2021) 9:1427. doi: 10.3390/healthcare9111427, PMID: 34828474 PMC8623963

[10] GOV.UK (www.gov.uk). Social prescribing: applying all our health. (2022). Available at: https://www.gov.uk/government/publications/social-prescribing-applying-all-our-health/social-prescribing-applying-all-our-health (Accessed March 3, 2023).

[11] Husk K Blockley K Lovell R Bethel A Lang I Byng R. What approaches to social prescribing work, for whom, and in what circumstances? A realist review. Health Soc Care Community. (2020) 28:309–24. doi: 10.1111/hsc.12839, PMID: 31502314 PMC7027770

[12] Coventry PA Brown JE Pervin J Brabyn S Pateman R Breedvelt J. Nature-based outdoor activities for mental and physical health: systematic review and meta-analysis. SSM Popul Health. (2021) 16:100934. doi: 10.1016/j.ssmph.2021.100934, PMID: 34646931 PMC8498096

[13] Smith SM Wallace E O’Dowd T Fortin M. Interventions for improving outcomes in patients with multimorbidity in primary care and community settings. Cochrane Database Syst Rev. (2021) 10. doi: 10.1186/s13643-021-01817-z, PMID: 33448337 PMC8092473

[14] Kaypa Hoito. Multimorbidity (Monisairas potilas) current care guidelines. Available at: www.kaypahoito.fi: Suomalainen Lääkäriseura Duodecim; (2021).

[15] Strombom Y Magnusson P Karlsson J Fredrikson M. Health-related quality of life among frequent attenders in Swedish primary care: a cross-sectional observational study. BMJ Open. (2019) 9:e026855. doi: 10.1136/bmjopen-2018-026855, PMID: 31366640 PMC6678018

[16] Keyes CL Simoes EJ. To flourish or not: positive mental health and all-cause mortality. Am J Public Health. (2012) 102:2164–72. doi: 10.2105/AJPH.2012.300918, PMID: 22994191 PMC3477942

[17] Appelqvist-Schmidlechner KTK Tamminen N Nordling E Solin P. Mitä on positiivinen mielenterveys ja kuinka sitä mitataan? Suom Laakaril. (2016) 71:1759–64.

[18] Appleton SL Melaku YA Reynolds AC Gill TK de Batlle J Adams RJ. Multidimensional sleep health is associated with mental well-being in Australian adults. J Sleep Res. (2022) 31:e13477. doi: 10.1111/jsr.13477, PMID: 34622511

[19] Ropponen A Silventoinen K Hublin C Svedberg P Koskenvuo M Kaprio J. Sleep patterns as predictors for disability pension due to low back diagnoses: a 23-year longitudinal study of Finnish twins. Sleep. (2013) 36:891–7. doi: 10.5665/sleep.2718, PMID: 23729932 PMC3649831

[20] Khachatryan SG. Insomnia burden and future perspectives. Sleep Med Clin. (2021) 16:513–21. doi: 10.1016/j.jsmc.2021.05.006, PMID: 34325827

[21] Ge L Guyatt G Tian J Pan B Chang Y Chen Y. Insomnia and risk of mortality from all-cause, cardiovascular disease, and cancer: systematic review and meta-analysis of prospective cohort studies. Sleep Med Rev. (2019) 48:101215. doi: 10.1016/j.smrv.2019.101215, PMID: 31630016

[22] Sipoo Demographics. (2022). Available at: https://www.sipoo.fi/organisaatio/avainluvut/2022 (Accessed December 31, 2021).

[23] Komppula B. Karppinen T. Virta H. Sundström A.-M. Ialongo I. Korpi K.. Ilmanlaatu Suomessa ilmanlaatumittausten ja satelliittihavaintojen perusteella. Finnish Meteorologica Institute, FMI publications (2021). Available at: http://hdl.handle.net/10138/334054

[24] Tyrväinen L. Savonen E.-M. Simkin J. Kohti suomalaista terveysmetsän mallia. Luonnonvara- ja biotalouden tutkimus (2017) 11:21.

[25] Matila A Koistinen A Lahti E. Metsä terveyden ja hyvinvoinnin edistäjänä—väliraportti. Tapio oy. (2018) 1–30.

[26] Heikkinen M. Hälsofrämjande Hälsoskog gruppverksamhet i Sibbo kommuns social-och hälsovårdstjänster—upplevelser ur ett deltagarperspektiv. (2022). Available at: https://urn.fi/URN:NBN:fi:amk-2022121429968

[27] Tennant R Hiller L Fishwick R Platt S Joseph S Weich S. The Warwick-Edinburgh mental well-being scale (WEMWBS): development and UK validation. Health Qual Life Outcomes. (2007) 5:63. doi: 10.1186/1477-7525-5-63, PMID: 18042300 PMC2222612

[28] Taggart F. S.-B. Parkinson S. Warwick-Edinburgh Mental Well-being Scale (WEMWBS) User Handbook (2019). Available at: https://s3.amazonaws.com/helpscout.net/docs/assets/5f97128852faff0016af3a34/attachments/5fe10a9eb624c71b7985b8f3/WEMWBS-Scale.pdf

[29] Shah N Cader M Andrews WP Wijesekera D Stewart-Brown SL. Responsiveness of the short Warwick Edinburgh mental well-being scale (SWEMWBS): evaluation a clinical sample. Health Qual Life Outcomes. (2018) 16:239. doi: 10.1186/s12955-018-1060-2, PMID: 30577856 PMC6303870

[30] Maheswaran H Weich S Powell J Stewart-Brown S. Evaluating the responsiveness of the Warwick Edinburgh mental well-being scale (WEMWBS): group and individual level analysis. Health Qual Life Outcomes. (2012) 10:156. doi: 10.1186/1477-7525-10-156, PMID: 23270465 PMC3560098

[31] Smith ORF Alves DE Knapstad M Haug E Aaro LE. Measuring mental well-being in Norway: validation of the Warwick-Edinburgh mental well-being scale (WEMWBS). BMC Psychiatry. (2017) 17:182. doi: 10.1186/s12888-017-1343-x, PMID: 28499368 PMC5427526

[32] Cole RJ Kripke DF Gruen W Mullaney DJ Gillin JC. Automatic sleep/wake identification from wrist activity. Sleep. (1992) 15:461–9. doi: 10.1093/sleep/15.5.461, PMID: 1455130

[33] Koponen P. Borodulin K. Lundqvist A. Sääksjärvi K. Koskinen S. Terveys, toimintakyky ja hyvinvointi Suomessa: FinTerveys 2017-tutkimus. THL (2018).

[34] Huhtakangas M Kyngas H Bloigu R Kanste O. Differentiating middle-aged long-term and short-term frequent attenders by means of the northern Finland birth cohort 1966 study. Scand J Caring Sci. (2021) 35:813–23. doi: 10.1111/scs.12896, PMID: 32740948

[35] Suvisaari J. Appelqvist-Schmidlechner K. Solin P. Ristiluoma N. Pietilä A. Koskinen S.. Psyykkisen kuormittuneisuuden ja positiivisen mielenterveyden muutokset suomalaisessa aikuisväestössä vuosina 2017–2020: FinTerveys 2017-seurantatutkimuksen tuloksia. Tutkimuksesta tiiviisti 36/2021. Terveyden ja hyvinvoinnin laitos, Helsinki (2021).

[36] World Health Organization. Comprehensive mental health action plan 2013–2030. (2021). Available at: https://www.who.int/publications/i/item/9789240031029

[37] Stier-Jarmer M Throner V Kirschneck M Immich G Frisch D Schuh A. The psychological and physical effects of forests on human health: a systematic review of systematic reviews and meta-analyses. Int J Environ Res Public Health. (2021) 18:770. doi: 10.3390/ijerph18041770, PMID: 33670337 PMC7918603

[38] Freedman A Nicolle J. Social isolation and loneliness: the new geriatric giants: approach for primary care. Can Fam Physician. (2020) 66:176–82. PMID: 32165464 PMC8302356

[39] Lonergan-Cullum M Hooker SA Levy R Ricco J. A new pandemic of loneliness. J Am Board Fam Med. (2022) 35:593–6. doi: 10.3122/jabfm.2022.03.210461, PMID: 35641036

[40] Tester-Jones M White MP Elliott LR Weinstein N Grellier J Economou T. Results from an 18 country cross-sectional study examining experiences of nature for people with common mental health disorders. Sci Rep. (2020) 10:19408. doi: 10.1038/s41598-020-75825-9, PMID: 33159132 PMC7648621

[41] Maas J Van Dillen SM Verheij RA Groenewegen PP. Social contacts as a possible mechanism behind the relation between green space and health. Health Place. (2009) 15:586–95. doi: 10.1016/j.healthplace.2008.09.006, PMID: 19022699

[42] Astell-Burt T Feng X Kolt GS. Does access to neighbourhood green space promote a healthy duration of sleep? Novel findings from a cross-sectional study of 259 319 Australians. BMJ Open. (2013) 3:e003094. doi: 10.1136/bmjopen-2013-003094, PMID: 23943772 PMC3740246

[43] Yeon PS Jeon JY Jung MS Min GM Kim GY Han KM. Effect of Forest therapy on depression and anxiety: a systematic review and meta-analysis. Int J Environ Res Public Health. (2021) 18:685. doi: 10.3390/ijerph182312685, PMID: 34886407 PMC8657257

[44] Capaldi CA Dopko RL Zelenski JM. The relationship between nature connectedness and happiness: a meta-analysis. Front Psychol. (2014):976. doi: 10.3389/fpsyg.2014.00976, PMID: 25249992 PMC4157607

[45] Martyn P Brymer E. The relationship between nature relatedness and anxiety. J Health Psychol. (2016) 21:1436–45. doi: 10.1177/1359105314555169, PMID: 25370570

[46] Richardson M Passmore H-A Lumber R Thomas R Hunt A. Moments, not minutes: the nature-wellbeing relationship. Int J Wellbeing. (2021) 11:8–33. doi: 10.5502/ijw.v11i1.1267, PMID: 37548165

[47] Gascon M Triguero-Mas M Martinez D Dadvand P Rojas-Rueda D Plasencia A. Residential green spaces and mortality: a systematic review. Environ Int. (2016) 86:60–7. doi: 10.1016/j.envint.2015.10.013, PMID: 26540085

[48] Marselle MR Lindley SJ Cook PA Bonn A. Biodiversity and health in the urban environment. Curr Environ Health Rep. (2021) 8:146–56. doi: 10.1007/s40572-021-00313-9, PMID: 33982150 PMC8115992

[49] Hanski I von Hertzen L Fyhrquist N Koskinen K Torppa K Laatikainen T. Environmental biodiversity, human microbiota, and allergy are interrelated. Proc Natl Acad Sci. (2012) 109:8334–9. doi: 10.1073/pnas.1205624109, PMID: 22566627 PMC3361383

[50] Markevych I Schoierer J Hartig T Chudnovsky A Hystad P Dzhambov AM. Exploring pathways linking greenspace to health: theoretical and methodological guidance. Environ Res. (2017) 158:301–17. doi: 10.1016/j.envres.2017.06.028, PMID: 28672128

[51] Ulrich RS Simons RF Losito BD Fiorito E Miles MA Zelson M. Stress recovery during exposure to natural and urban environments. J Environ Psychol. (1991) 11:201–30. doi: 10.1016/S0272-4944(05)80184-7

[52] Kaplan R Kaplan S. The experience of nature: A psychological perspective. New York, NY: Cambridge University Press (1989).

[53] Lanki T Siponen T Ojala A Korpela K Pennanen A Tiittanen P. Acute effects of visits to urban green environments on cardiovascular physiology in women: a field experiment. Environ Res. (2017) 159:176–85. doi: 10.1016/j.envres.2017.07.039, PMID: 28802208

[54] Pasanen T Johnson K Lee K Korpela K. Can nature walks with psychological tasks improve mood, self-reported restoration, and sustained attention? Results from two experimental field studies. Front Psychol. (2018) 9:2057. doi: 10.3389/fpsyg.2018.02057, PMID: 30425671 PMC6218585

[55] Von Lindern E Lymeus F Hartig T. The restorative environment: a complementary concept for salutogenesis studies. In: Mittelmark MB Sagy S Eriksson M Bauer GF Pelikan JM Lindström B.. (editors), The Handbook Salutogen. Springer, Cham. (2017). 181–95. 28590625

[56] Prior A Vestergaard M Larsen KK Fenger-Gron M. Association between perceived stress, multimorbidity and primary care health services: a Danish population-based cohort study. BMJ Open. (2018) 8:e018323. doi: 10.1136/bmjopen-2017-018323, PMID: 29478014 PMC5855234

[57] Kujanpaa TS Jokelainen J Auvinen JP Timonen MJ. The association of generalized anxiety disorder and somatic symptoms with frequent attendance to health care services: a cross-sectional study from the northern Finland birth cohort 1966. Int J Psychiatry Med. (2017) 52:147–59. doi: 10.1177/0091217417720894, PMID: 28792287

[58] Simkin J Ojala A Tyrvainen L. The perceived Restorativeness of differently managed forests and its association with Forest qualities and individual variables: a field experiment. Int J Environ Res Public Health. (2021) 18:422. doi: 10.3390/ijerph18020422, PMID: 33430350 PMC7825791

[59] Sievänen T. Neuvonen M. Luonnon virkistyskäyttö 2010. In: *Working papers of the Finnish Forest Research Institute*. (2011).

[60] OECD. State of health in the EU, Finland: Country health profile 2019. Paris/Brussels: European Observatory on Health Systems and Policies (2019).

[61] Helliwell J. F. Huang H. Norton M. Goff L. Wang S. World happiness, trust and social connections in times of crisis. Available at: https://worldhappiness.report/ed/2023/world-happiness-trust-and-social-connections-in-times-of-crisis/#ranking-of-happiness-2020-2022. Sustainable Development Solutions Network. (2023).

[62] Health and Welfare Inequalities Available at: https://thl.fi/fi/web/hyvinvointi-ja-terveyserot/seuranta-ja-vaikuttavuus/vaikuttavuus-ja-kustannukset. Finnish Institute for Health and Welfare; (2021).

[63] Rolf Myhrman AA Siljander E. Social expenditure scenarios—Effects of health promotion and a presentation of the analysis model. Finland: Ministry of Social Affairs and Health (2009).

[64] Ek S. Gender differences in health information behaviour: a Finnish population-based survey. Health Promot Int. (2015) 30:736–45. doi: 10.1093/heapro/dat063, PMID: 23985248

[65] Welen Schef K Tornvall P Alfredsson J Hagstrom E Ravn-Fischer A Soderberg S. Prevalence of angina pectoris and association with coronary atherosclerosis in a general population. Heart. (2023). doi: 10.1136/heartjnl-2023-322345, PMID: 37225242 PMC10511980

[66] Lynch M Jones CR. Social prescribing for frequent attenders in primary care: an economic analysis. Front Public Health. (2022) 10:902199. doi: 10.3389/fpubh.2022.902199, PMID: 36311628 PMC9615419

[67] Hyvinvointilähete Uusi Toimintamalli Available at: https://www.sitra.fi/artikkelit/hyvinvointilahete-uusi-toimintamalli-yksilon-ja-koko-yhteison-hyvinvoinnin-vahvistamiseksi/. Sitra; (2022).

[68] Full KM Kerr J Grandner MA Malhotra A Moran K Godoble S. Validation of a physical activity accelerometer device worn on the hip and wrist against polysomnography. Sleep Health. (2018) 4:209–16. doi: 10.1016/j.sleh.2017.12.007, PMID: 29555136 PMC5863644

[69] de Zambotti M Cellini N Goldstone A Colrain IM Baker FC. Wearable sleep Technology in Clinical and Research Settings. Med Sci Sports Exerc. (2019) 51:1538–57. doi: 10.1249/MSS.0000000000001947, PMID: 30789439 PMC6579636

[70] Ministery of Finance. Sustainable Growth Programme for Finland. Finland; (2023) Available at: https://vm.fi/en/sustainable-growth-programme-for-finland.

[71] Hyvinvoinnin ja Terveyden Edistämisen Palvelukonsepti. Available at: https://thl.fi/fi/tutkimus-ja-kehittaminen/tutkimukset-ja-hankkeet/suomen-kestavan-kasvun-ohjelma-rrp-/hyvinvointia-ja-terveytta-edistavat-toimintamallit/hyvinvoinnin-ja-terveyden-edistamisen-palvelukonsepti. The Finnish Institute for Health and Welfare; (2023).

