# Supplementary material for: Targeted health promotion with guided nature walks or group exercise: a controlled trial in primary care
Source: Front Public Health. 2023 Aug 24;11:1208858. doi: 10.3389/fpubh.2023.1208858 (PMC10520711; doi:10.3389/fpubh.2023.1208858)
Supplement: Supplementary file 1 [file Table_1.DOCX]

Supplementary Material

Targeted health promotion with guided nature walks or group exercise: a controlled trial in primary care

Annika Kolster*, Malin Heikkinen, Adela Pajunen, Anders Mickos, Heini Wennman, Timo Partonen

*** Correspondence:** Corresponding Author: *Annika Kolster,* [*annika.kolster@helsinki.fi*](mailto:annika.kolster@helsinki.fi)

# Supplementary Data

# Intervention Programmes

**Description of Nature intervention**

*Time period and places*

Each programme consisted of 7 forest sessions within 8 weeks. The autumn programme started in September around the equinox when the weather is typically relatively warm post-summer and ended in the end of November in early winter conditions. In spring the programme started around mid-April in early spring conditions and ended in mid-June in summer conditions. The duration of each session varied depending on the place of a visit between 2 to 4 hours, the average duration being 2.8 hours.

Every session took part in a different natural environment; the idea was to introduce the participants not only to different ways of experiencing nature but also to a variety of sceneries to fit the needs of different psychological states. Variety of places also enabled participants to learn new routes in the area. 3 of the 7 excursions were in different locations of Sipoonkorpi National Park. The nature of Sipoonkorpi is a mixture of spruce (*Picea abies*) dominated forest areas, mires, and traditional agricultural landscapes. The biodiversity of Sipoonkorpi is very high. The topography of the area is varied, providing naturally both places of refuge and prospect. Other places visited included a nature reserve of rocky old-growth pine forest (*Pinus sylvestris*), a lake near a farm, a privately owned forest where it was possible to make a campfire and a vast sea landscape in the Archipelago of Porvoo.

*Activities and guides*

Participants were guided walking at a peaceful tempo and taking numeorus breaks. The covered distances varied between 1 and 4 kilometres, average being 2 km. Simple exercises, tailored according the places, were introduced to enhance the nature contact. They were for instance: listening to natural sounds, watching miniature landscapes with magnifying loupe, laying down in a moss carpet, observing wildlife, feeding ants and birds and finding one’s own favourite places. One of the autumn excursions took place in pitch dark, flashlights and oil lanterns were used to get around. During spring, edible plants were identified and collected.

. A new aspect of nature experience was introduced each time:

- sensing specificities of a place
- activating senses
- relaxation in nature
- experiencing beauty
- empowerment through perception of natural elements
- finding one’s way and coping in nature
- noticing nature’s role in one’s life

Two professional guides, one biologist and one geographer, guided the groups. Knowledge about nature and the ways nature can revitalize us, for example., about the role of beneficial microbes and clean air was offered buta special emphasis was put on non-conceptual experience.

**Description of the Sports intervention**

The programme used in the intervention was planned to be as alike as the that in the health forest regarding duration (mostly 2 h) and amount and intensity of physical exercise. The programme was planned and executed by professional sports leaders Meetings lasted 2 to 3.5 h and took place in the afternoon (17:00 to 19:00 hours) and on one Saturday morning.

Participants were provided with information about health benefits of PA and were introduced to a wide variety of sports. Each meeting had a theme. The order of themes varied.

- Muscle training and relaxation (indoor activity) Introduction of the programme
- Aquatic workout (indoor swimming pool) Theme: try the power and softness of the water
- Mindfulness/yoga (Indoor activity) Theme: towards a more relaxed and de-stressed life
- City orientation, Porvoo Theme: outdoor activities in cultural small city surrounding (duration 3.5 h)
- Nordic walking and muscle training (outdoor) Theme: introducing outdoor activities and tools for outdoor exercise
- Exercise individually and together (indoor activity) Theme: try the game kin-ball
- Easy Olympic Games, the central sports field (outdoor) Theme: exercise and fun
